# Functional Polymeric Systems for Advanced Industrial Applications

**DOI:** 10.3390/polym15051277

**Published:** 2023-03-02

**Authors:** Huacheng Zhang

**Affiliations:** School of Chemical Engineering and Technology, Xi’an Jiaotong University, Xi’an 710049, China; zhanghuacheng@xjtu.edu.cn

Functional polymeric systems constitute a huge family of novel hierarchical architectures categorized by different polymeric shapes, such as linear, brush-like, star-like, dendrimer-like and network-like ones; various components, such as organic–inorganic hybrid oligomeric/polymeric materials and metal-ligated polymers; different features, such as porous polymers; and diverse approaching strategies and driving forces, such as conjugated/supramolecular/mechanical force-based polymers and self-assembled networks [1]. Current academic studies of functional polymeric systems not only focus on the development of synthesis methods, such as general polymerization, organic and supramolecular approaches, but also investigate multi-dimensional morphologies and particular physiochemical properties [1], which pave the way to practical and advanced industrial applications. For example, in [2], the design and preparation of fabricated morphologies, such as hierarchical dendrite, clearly exhibited enhanced surface area and improved applicable behaviors in specialized fields compared to pristine ones. Thus, an in-depth understanding of the chemical, physical and instrumental characterization, manufacturing and advanced industrial applications of functional polymeric systems/materials is highly relevant to their performance and development.

Functional polymeric systems have served diverse roles in advanced applications. For example, in [3], polymers such as polyvinyl alcohol served as the substrate for loading coronene tetra-carboxylate salts to fabricate interesting room-temperature afterglow materials in a series of applications in imaging, lighting and therapy. In addition, the preparation of proper polymeric architecture is significant in enhancing the physiochemical properties of other functional materials. For example, in [4], Zhang et al. found an interesting approach to stabilizing wood via the in situ construction of thermotropic shape memory polymers such as methyl methacrylate and polyethylene glycol diacrylate containing a cross-linked copolymer network inside the wood structures.

In particular, due to instinctive stability, biocompatibility and flexibility, functionalized polymeric systems have been widely applied to biomedicines. For example, in [5], Do et al. investigated biomedical applications in the treatment of severe burn injury and other infection-related skin complications. They found that a polycaprolactone–gelatin–silver membrane can electively kill both Gram-positive and Gram-negative bacteria with reasonable minimum bactericidal concentration, exhibiting a bactericidal effect during the first 24 h. In addition, in [6], Nguyen et al. prepared methoxy polyethylene glycol modified polyamidoamine dendrimer generation 3.0 and discovered that it could prolong the release of retrovirus drugs and effectively enhance the treatment of human immunodeficiency viruses.

A great deal of work has been accomplished in applying functional polymeric systems; however, there is still much to be done to effectively promote cooperation between academic studies and practical engineering applications. For future studies in this area, we would like to call more attentions to academic studies related to the theory and simulation of polymeric materials, as well as industrial considerations in the advanced manufacturing, processing and performance of polymeric materials. Additionally, it is also significant to expand the advanced industrial applications of functional polymeric systems to novel sensor and electronic devices, supramolecular hydrogels and smart materials, the storage and adsorption of significant molecules and novel energy solutions.
